# Increasing Donor Endothelial Cell Pool by Culturing Cells from Discarded Pieces of Human Donor Corneas for Regenerative Treatments

**DOI:** 10.1155/2019/2525384

**Published:** 2019-07-21

**Authors:** Mohit Parekh, Vito Romano, Alessandro Ruzza, Stephen B. Kaye, Diego Ponzin, Sajjad Ahmad, Stefano Ferrari

**Affiliations:** ^1^Institute of Ophthalmology, University College London, London, UK; ^2^International Center for Ocular Physiopathology, Fondazione Banca degli Occhi del Veneto Onlus, Venice, Italy; ^3^St. Paul's Eye Unit, Royal Liverpool Broadgreen University Hospital, Liverpool, UK; ^4^Instituto Universitario Fernandez-Vega, Universidad de Oviedo and Fundacion de Investigacion on Oftalmologica, Oviedo, Spain; ^5^Institute of Ageing and Chronic Disease, Department of Eye and Vision Science, University of Liverpool, Liverpool, UK; ^6^Moorfields Eye Hospital NHS Trust Foundation, London, UK

## Abstract

**Purpose:**

To investigate if the peripheral corneal endothelium that is usually discarded after a corneal transplant could be used for endothelial cell culture.

**Methods:**

Donor corneas (*n* = 19) with a mean age of 72 years, male : female ratio of 15 : 4, and death-to-preservation time of 10 hours were assessed for endothelial cell density (ECD) and number of dead cells before isolation. Alizarin red staining (*n* = 3) was performed to check the morphology of cells in the center and periphery. Descemet's membrane-endothelial complex was peeled from the center (8.25 mm) and the periphery (2.75 mm) and plated in two different wells of an 8-well chamber slide with media refreshed every alternate day. The confluence rate was monitored by microscopy. Live/dead analysis was performed (*n* = 3) at confluence. Tag-2A12 as a monoclonal antibody against peroxiredoxin-6 (Prdx-6) (*n* = 4), ZO-1 (zonula occludens-1) as a tight junction protein (*n* = 4), and Ki-67 as a proliferative cell marker (*n* = 4) were used to characterize the cells at confluence.

**Results:**

At confluence, 8.25% average increase in the number of cells was observed from the central zone compared with 16.5% from the peripheral zone. Proliferation rate, hexagonality, Ki-67 positivity, and the cell area did not significantly differ between the groups (*p* > 0.05). All the proteins corresponding to the biomarkers tested were expressed in both the groups.

**Conclusions:**

Although there are significantly fewer amounts of peripheral cells available after graft preparation for keratoplasty, these cells can still be used for endothelial cell culture due to their proliferative capability. The peripheral cells that are discarded after graft preparation can thus be utilized to increase the donor endothelial cell pool for regenerative treatments.

## 1. Introduction

Human corneal endothelium is the posterior monolayer of hexagonal cells that are derived from the neural crest [1, 2]. They are nonproliferative *in vivo* due to their mitotic arrest [3] and show age-related decrease in cell density [4, 5]. The health and viability of these cells must be maintained to preserve the hydration and transparency of the cornea. According to the 2016 statistical report of Eye Bank Association of America (EBAA), approximately 40% of the keratoplasties performed in the United States were because of endothelial dysfunction. A global survey reported that despite several thousand keratoplasties taking place each year, 12 million patients are still on the waiting list for a corneal transplant [6]. Hence, it becomes necessary to identify alternatives that could reduce the demand of human donor corneas. Several attempts have been made to isolate and propagate human corneal endothelial cells (HCEnCs) and reviewed [7–11]. One successful clinical study has been reported for the treatment of bullous keratopathy [12] using cultured cells, so far. These studies have been performed utilizing tissues from young donors. However, most of the corneas from young donors are transplanted because of high endothelial cell counts. Thus, only old age donor tissues mostly remain available for cell culture or research.

It has been observed that during keratoplasties like penetrating keratoplasty (PK), Descemet stripping automated endothelial keratoplasty (DSAEK), or Descemet membrane endothelial keratoplasty (DMEK) (including preloaded DMEK) [13, 14], only the central (usually 7.5 mm–8.5 mm) zone is used for transplant and the remaining peripheral tissue is discarded. These discarded peripheral wastes contain a rich zone of putative stem cells [15]. Several studies have shown that there are more number of cells in the peripheral endothelium that contain higher proliferative potential [16–21]. Due to the size of the central zone used in current keratoplasty procedures, the discarded peripheral endothelial tissues could be useful in isolating and culturing the cells. Therefore, we set out to investigate if the peripheral endothelial cells that are usually discarded after surgeries like DMEK could be used for endothelial cell culture, as this may substantially increase the donor endothelial cell pool for regenerative treatments.

## 2. Materials and Methods

### 2.1. Ethical Statement

Corneal tissues were collected by the Veneto Eye Bank Foundation (FBOV, Italy) with written consent from the donor's next-of-kin to be used for research purposes under the guidelines and laws of Centro Nazionale di Trapianti, Rome, Italy. The tissues were unsuitable for transplantation due to the low endothelial cell count (<2200 cells/mm^2^). No other complications or indications were registered.

### 2.2. Endothelial Cell Count and Donor Characteristics

Average age, postmortem time, and gender of all the tissues (*n* = 19) were recorded retrospectively. The tissues were preserved in Cornea Max (Eurobio, Paris, France) at 31°C. ECD and dead cells were assessed by topical application of 100 *μ*L of 0.25% trypan blue (TB) stain (Thermo Fisher Scientific, Rochester, NY, USA), on the corneal endothelium for approximately 20 seconds and washed with phosphate buffered saline (1X PBS). Dead cells were recorded as the percentage of trypan blue positive cells (TBPCs). ECD and TBPCs were recorded before isolation on the cornea using a calibrated inbuilt eyepiece reticule for the inverted microscope (Axiovert, Zeiss, Germany), both in the center and at the periphery. The eyepiece reticule (10 × 10) was also used to check the number of cells at confluence and to calculate the proliferation rate. Average of 5 readings was taken from the central area and 3 readings from the peripheral area.

### 2.3. Alizarin Red

The corneal tissues (*n* = 3) were washed removing all the media remnants. The tissues were placed on a vacuum block with the endothelium facing the air. Approximately 250 *μ*L of alizarin red stain (Sigma-Aldrich) was added on the endothelium, incubated at room temperature (RT) for 3.5 minutes, and washed several times with PBS to remove the excess alizarin red stain. The tissues were placed in 1.8% sucrose solution with epithelium facing the air. The endothelial cells were viewed and imaged at different areas using an inverted microscope, both in the center and at the periphery.

### 2.4. Peeling Method

**Figure 1:**
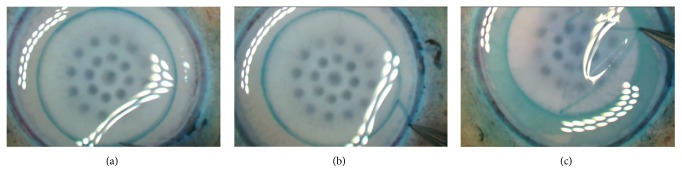
Peel and digest method. (a) After fixing the tissue on a vacuum block, an 8.25 mm Moria trephine (Moria, Antony, France) was used to gently tap the endothelial side on its surface to create a superficial cut, which was identified using trypan blue staining. (b) The peripheral 2.75 mm zone was gently detached using 120 mm acute forceps (e.janach, Como, Italy), (c) followed by peeling the central 8.25 mm zone.

The corneal tissues (*n* = 16) were washed in sterile PBS. 8.25 mm Moria trephine (Moria, Antony, France) was used to gently tap the endothelial side creating a superficial cut. It was assured that the blade trephined an 8.25 mm zone as centrally as possible. The cut mark was identified using trypan blue stain (Figure 1(a)). The peripheral 2.75 mm zone was gently peeled using 120 mm acute forceps (e.janach, Como, Italy) (Figure 1(b)) followed by peeling the central 8.25 mm zone (Figure 1(c)).

### 2.5. Media Formulation

The cell culture medium was a mixture of HamF12 and M199 (Sigma-Aldrich, St. Louis, Missouri, USA) (1 : 1), supplemented with 5% FBS (Sigma-Aldrich, St. Louis, Missouri, USA), 1% ascorbic acid (Sigma-Aldrich, St. Louis, Missouri, USA), 0.5% Insulin-Transferrin-Selenium (ITS) (Thermo Fisher Scientific, Waltham, Massachusetts, USA), recombinant human FGF basic (10 ng/mL) (Thermo Fisher Scientific, Waltham, Massachusetts, USA), 10 *μ*M ROCK inhibitor (Y-27632) (Miltenyi Biotec, Bergisch Gladbach, Germany), and 1% PenStrep (Thermo Fisher Scientific, Waltham, Massachusetts, USA) [9–11].

### 2.6. Isolation of Cells

The central and peripheral tissue pieces were incubated in 2 mg/mL collagenase type 1 diluted in human endothelium-SFM supplemented with 5% FBS and 1% PenStrep (Thermo Fisher Scientific, Rochester, NY, USA) solution for 2 hours at 31°C and 5% CO_2_. The cells collected with collagenase were centrifuged for 5 minutes at 1000 rpm. The supernatant was removed, and the cells were resuspended with TrypLE express (1X) (Life Technologies, Monza, Italy) for 10 minutes at 37°C to obtain single cells. The cells were resuspended with 200 *μ*L of the cell culture media after counting them using a haemocytometer slide.

### 2.7. Plating

All the chambers of Lab-Tek II chamber slides (8 chambers, 25 × 75 mm, 0.7 cm^2^ culture area) from Thermo Fisher Scientific (Rochester, NY, USA) were coated with 50 *μ*L of FNC coating mix (cell attachment reagent (FNC Coating Mix) BRFF AF-10, US Biological Life Sciences, Salem, Massachusetts, USA) for 30 minutes at 37°C and 5% CO_2_. The residual coating was removed before plating the cells. 100 *μ*L of final cell suspension was prepared from both the central and peripheral cells of each donor cornea. The cell suspension from the central cornea was mixed well and added in one chamber of the 8-well chamber slide and, similarly, the peripheral cells in another chamber of the slide. The cells were not passaged for this study. The “number of cells plated” was recorded for all the cultures.

### 2.8. Morphological Analysis

The cells from the tissues (*n* = 15) were monitored and visualized every alternate day until confluence using a 10 × 10 reticule (0.1 mm^2^) attached to the eyepiece of an inverted microscope (Axiovert, Zeiss, Germany). The proliferation rate was monitored by counting the number of boxes filled with the cells and recorded every alternate day manually using an eyepiece reticule as described above, till confluence.

### 2.9. Live/Dead Assay

The cultured cells (*n* = 3) were washed with PBS prior to the assay. 3 *μ*L of Hoechst 33342 (H) (Thermo Fisher Scientific, Rochester, NY, USA), 2 *μ*L of ethidium homodimer (EthD-1) (E), and 2 *μ*L Calcein AM (C) (live/dead viability/cytotoxicity kit, Thermo Fisher Scientific, Rochester, NY, USA) were mixed in 1 mL of PBS. 100 *μ*L of the final solution was directly added on the cultured cells and incubated at RT in the dark for 30 minutes. With the solution still on the cells, HEC was viewed and imaged within 1 hour using a Nikon Eclipse Ti-E (Nikon, Burgerweeshuispad, Amsterdam) microscope with NIS Elements software (Nikon) [22].

### 2.10. Immunostaining

The cultured cells were washed with PBS and fixed in 4% paraformaldehyde (PFA) at RT for 20 minutes. The cells were permeabilized with 0.5% Triton X-100 in PBS for 30 minutes (note: the permeabilization step was not performed for 2A12 considering that it is a cell surface marker and it may damage the epitope). After blocking with 10% bovine serum albumin (BSA) for 2 hours at RT, the cells were incubated overnight at 4°C with primary antibodies such as anti-Ki-67 (*n* = 4) (1 : 200 (MIB-1, Milan, Italy)) and anti-2A12 (*n* = 4) (1 : 100 (Tag-2A12)) (Bioprocessing Technology Institute, Singapore) (note: anti-ZO-1 (*n* = 4), 1 : 200 (ZO1-1A12, Alexa Fluor 488, Thermo Fisher Scientific, Rochester, NY, USA), was only incubated for 3 hours at RT, washed, and analyzed microscopically as it was already conjugated with Alexa Fluor). The samples (except ZO-1) were incubated with goat anti-mouse fluorescein isothiocyanate- (FITC-) conjugated secondary antibody in 5% BSA for 2 hours at RT. After each step, the cells were washed 3 times with 1X PBS. After removing the wall of the Lab-Tek slide, the cells were covered with mounting medium containing 4′,6-diamidino-2-phenylindole (DAPI) and covered with the cover slips. The Nikon Eclipse Ti-E (Nikon, Burgerweeshuispad, Amsterdam) microscope was used with NIS Elements software (Nikon) to observe the expression of the proteins and obtain images.

### 2.11. Measurements and Statistical Analysis

ImageJ (FIJI) software was used to measure and analyze the data. For ZO-1, the area was selected and using predefined commands in Macros for converting the image to overlay masks, the total number of cells was automatically counted. The hexagonal and polymorphic cells were counted based on the cell structure in the particular area (with 6 borders for hexagonal cells and less than 4 borders for polymorphic cells). The Macros was designed to obtain results by inserting an algorithm in the ImageJ analysis.

The cell surface area was determined using Calcein AM by splitting the channels and selecting a cell with a freehand tool followed by measuring the area with size limits of 150–10,000 *μ*m^2^.

For Ki-67 positivity, the particles were analyzed using an outline option to convert the image, and watershed was applied if necessary. The percentage of Ki-67 positive cells was counted based on the number of particles observed in the area compared with the number of cells counted using nuclei staining.

The nonparametric Wilcoxon test and Student's *t*-test for paired data using SAS statistical software were employed to check the statistical significance between the central and peripheral cells, where *p* < 0.05 was deemed significant. A post hoc correction to the significance was applied using the Bonferroni test.

## 3. Results

### 3.1. Donor Characteristics and Cell Numbers

**Figure 2:**
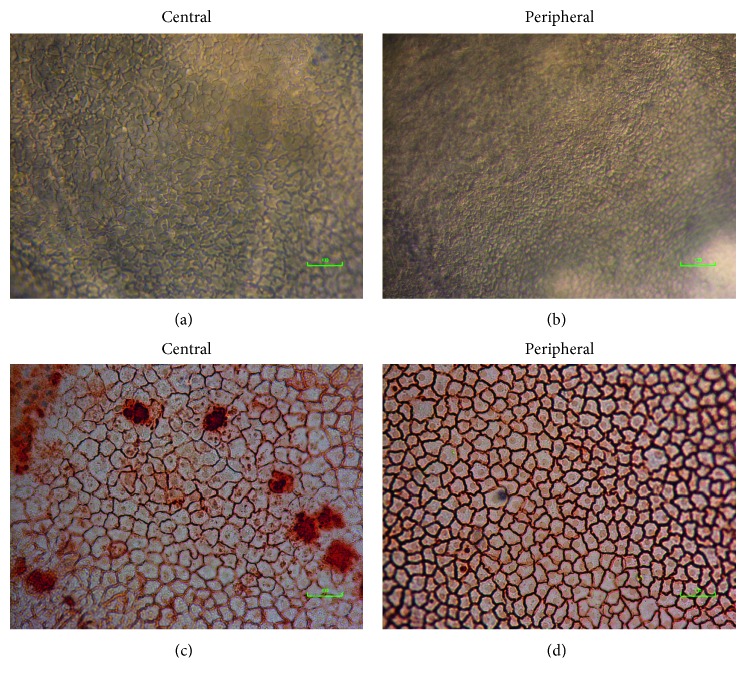
Morphology of the endothelial cells on the corneal tissues using different staining methods. (a) ECD count in the central region was lower compared (scale: 100 *μ*m) with (b) ECD in the periphery (scale: 250 *μ*m). (c) Morphology in the center of the cornea (scale: 100 *μ*m) compared with (d) that in its periphery (scale: 100 *μ*m) was observed using alizarin red staining.

Donor corneas (*n* = 19) with a mean age of 71.57 ± 5.4 years, male : female ratio of 15 : 4, and death-to-preservation time of 10.45 ± 5.2 hours were used for this study. On the tissues, ECD in the center (Figure 2(a)) and the periphery (Figure 2(b)) was observed and recorded. 8.71% (approximately 150 cells/mm^2^) average increase in the number of cells was found from the peripheral zone compared with the central zone (*p* < 0.05). Isolated HCEnCs from donors (*n* = 1) that did not reach confluence were excluded from this study. A morphological difference was observed in the cells from the central zone (Figure 2(c)) of the cornea compared with those at the periphery (Figure 2(d)) when viewed using alizarin red staining. At confluence, 8.25% average increase in the number of cultured cells was observed from the central zone compared with 16.5% average increase from the peripheral zone.

### 3.2. Proliferation Rate

**Figure 3:**
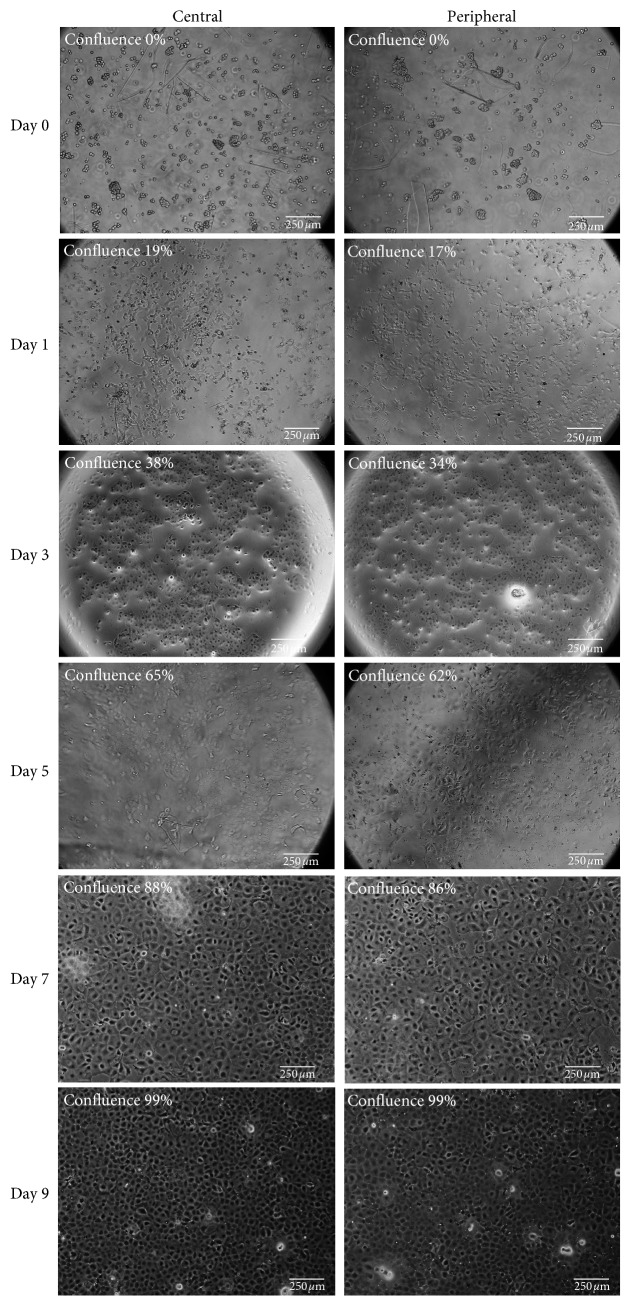
Morphology and confluence rate of the HCEnCs at alternate days of culture.

HCEnCs were observed every alternate day (Figure 3) for morphology, growth pattern, and proliferation rate. The average proliferation rate (%) at day 0, 1, 3, 5, 7, and 9 between central and peripheral endothelial cells was 0, 19, 38, 65, 88, and 99, and 0, 17, 34, 62, 86, and 99, which was not found to be statistically significant (*p*=0.9339) (*n* = 15).

### 3.3. Viability Staining (HEC) and Expression of PRDX-6 (2A12), ZO-1, and Ki-67

**Figure 4:**
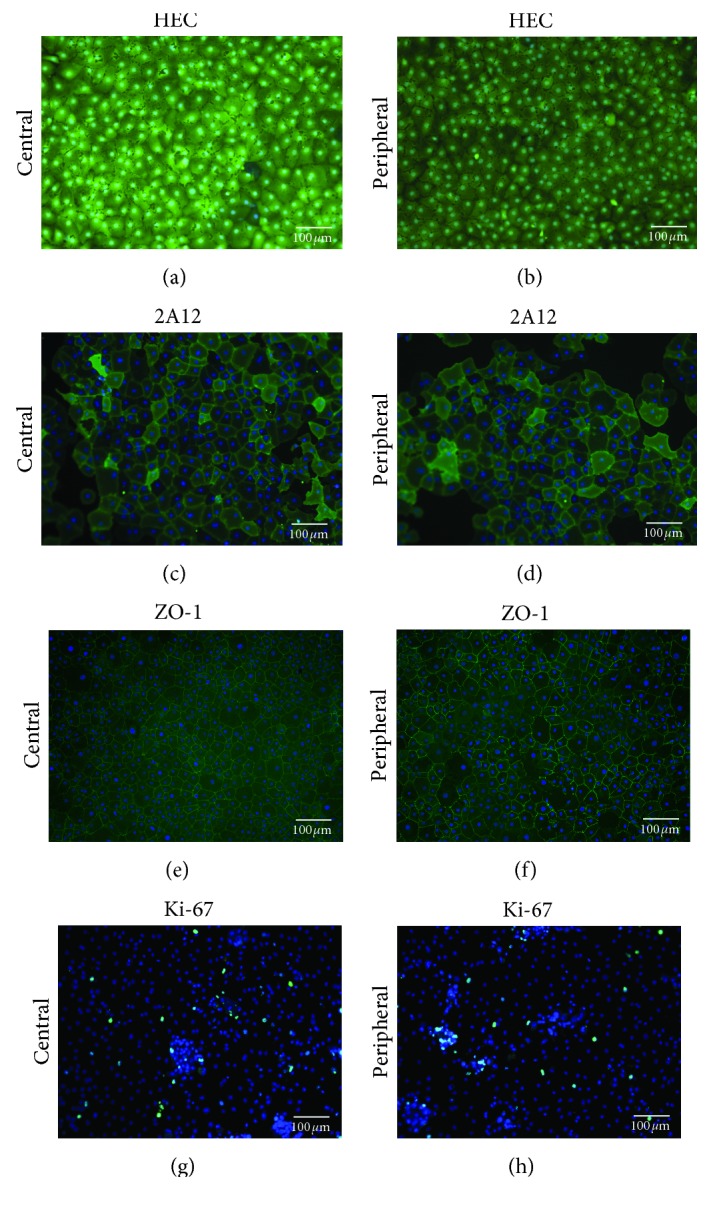
Live/dead analysis using HEC staining. Cell viability was observed in the (a) central and (b) peripheral zone. Expression of PRDX-6 (Tag-2A12) was observed in the cells cultured from (c) the center of the cornea and from (d) its periphery. ZO-1 was expressed in the cells from (e) the center and from (f) the periphery. Ki-67 was expressed in (g) the center and (h) the periphery.

100% cell viability (Calcein AM positivity) was observed in both the groups without any positivity of ethidium homodimer stain, which stains the dead cells. From the morphological observation, cell sizes of HCEnCs from the center (Figure 4(a)) were slightly larger than HCEnCs from the periphery (Figure 4(b)); however, the difference was not significant. Expression of PRDX-6 (Tag‐2A12) was observed in the cells cultured from the central zone (Figure 4(c)) and from the periphery of the cornea (Figure 4(d)). ZO-1 was expressed in the cells from the center (Figure 4(e)) and from the periphery (Figure 4(f)). Ki-67 was expressed in both the groups (Figure 4(g): central; Figure 4(h): periphery).

### 3.4. Morphology of Cultured Cells

The cell area was determined on 20 cells per condition using Calcein AM staining and ImageJ analysis. Difference in the cell area (*μ*m^2^) between the center and the peripheral group was not found to be statistically significant (*p*=0.8714), as listed in Table 1. No significant difference was found in the percentage of hexagonality between the cultured center and peripheral cells (*p*=0.5593). The cells did not show statistical significance in terms of polymorphism (*p*=0.7434). Ki-67 was expressed in both the groups without any statistical significance (*p*=0.5813). The values are listed in Table 1.

## 4. Discussion

Replacing the diseased cells of the recipient with that of the healthy corneal endothelial cells from a cadaveric donor through common keratoplasty procedures like PK/EK is the current treatment option for treating endothelial dysfunction. However, for these keratoplasty techniques, there is a huge demand of healthy human corneal donor tissues that are difficult to obtain due to limited supply. Therefore, alternative treatment options such as HCEnC propagation and transplantation could play an important role as tissue replacements [23, 24].

In terms of number of cells, Schimmelpfennig [21] and Daus et al. [18] reported a significant increase in the peripheral endothelial cell density (ECD) compared with the central ECD. However, Amann et al. showed regional differences in ECD counts between central, paracentral, and peripheral ECD in normal human corneas [16]. Regional differences in proliferative capacity have also been studied from young (younger than 30 years) and old-aged (older than 50 years) donor corneas [19]. It is also shown that HCEnCs cultured from the central and peripheral regions of a single donor grow in a similar manner [19]. However, Konomi et al. did not study the proliferative capacity of the cells from far periphery (9.5 mm–11.0 mm), which is available after every suitable graft for transplant. Another study also indicated that HCEnCs cultured from central and peripheral regions retain proliferative capacity [20]. Bednarz et al. however showed that HCEnCs from the peripheral area are able to replicate, but cells from the center exhibit little to no mitotic activity [17]. Indeed, it has been observed that cultivated HCEnCs derived from old donor tissues have lower proliferative capability, a senescent cell phenotype, with enlarged cellular morphology, which may in turn affect overall cell yield as well as its inherent functional ability [25]. Old donor tissues, i.e., above 65 years of age, are more frequently available for research as most get rejected due to lower endothelial cell count that is required for transplantation (data from FBOV, Italy 2016 annual report) [26]. Expansion and culturing of HCEnCs from old donor tissues (from the center and periphery separately) therefore could be advantageous as it could reduce the waiting time for a suitable primary source of endothelial cells.

During organ culture preservation, if the tissues are found unsuitable for transplantation due to poor ECD values then they are generally discarded or used for research, if consented. Moreover, from our previous study, we have noticed that tissues obtained from organ culture media show successful cell culture [22]. Hence, for this study, we used the tissues directly from organ culture storage media without deswelling the tissue.

We have observed that the peripheral cells have a high proliferative capability. Although the number of plated cells from the peripheral zone was significantly lower than those from the central zone, the cells did not show any statistical difference in terms of characterization and parameters that were checked. Considering that a normal human cornea is 11 mm (cell area of 94.985 mm^2^) in diameter and a routine DMEK graft is 8.25 mm (cell area of 53.429 mm^2^) in diameter, the peripheral tissue of 2.75 mm (cell area of 41.556 mm^2^) is wasted per DMEK, depending on the type of surgery that takes place with specific diameters. For example, assuming that in a normal DMEK graft, the number of cells counted is 2500 cells/mm^2^, which is roughly 133,572 cells in an 8.25 mm diameter graft (i.e., area of the graft (53.429 mm^2^) × number of cells/mm^2^). Considering a general 10% increase of cells in the periphery of the same tissue, the number of cells counted will be 2750 cells/mm^2^, which is roughly 114,279 cells in the remaining 2.75 mm peripheral area (i.e., area of the graft (41.556 mm^2^) × number of cells/mm^2^). The difference observed between the central and the peripheral cells would therefore be approximately 19,300 less number of cells plated from the peripheral zone. In our study, we found an average of 14,510 cells (this value excludes the number of dead cells) plated less from the peripheral zone compared with those from the central zone, which is nearing the hypothetical value of 19,300 cells. At confluence, the difference between the number of cells from the central and peripheral zone was approximately 200 cells/mm^2^. However, as the increase in the number of cells was significantly higher from the peripheral zone, it seems that both the zones can be cultured up to confluence.

Earlier, mostly PK was performed but with recent advances in the EK procedures, it is now possible to utilize the far periphery of the endothelium for cell isolation and proliferation in vitro. Therefore, from this study, we found two important advantages of culturing a central and/or a peripheral graft:

- The usually discarded peripheral tissues after surgeries like PK, DSAEK, or DMEK could be used in cell culture facilities to grow another graft and use it for transplantation. The final cell density at confluence shows a value similar to the transplanted graft as the proliferative capacity of the peripheral cells is higher.
- Two complete endothelial grafts can be generated from a single donor tissue (research grade) discarded from the transplant due to poor endothelial cell density.

Moreover, as the tissues obtained in this study were from old age donors, the primary endothelial cell pool increases due to the discard rate of old age donor tissues. Our protocol mimics that of the currently used DMEK graft sizes; therefore, it may also be easy to retrieve these cells without any further routine modifications in the eye bank or in the theatre increasing the donor endothelial cell pool, as the peripheral 2.75 mm zone is rich in proliferative cells [26].

This study could also open up possibilities of harvesting cells from patients with small central endothelial decompensation, who still have some good cells at the periphery, to culture and transplant them as an autograft and, by that, reducing the chances of endothelial rejection. Although there will be two surgical procedures required, if the central corneal endothelium is able to maintain and not worsen the edema, then the peripheral cells can be cultured to confluence and used as an autograft. We have also showed that peripheral tissues deemed for preloaded DMEK could successfully be preserved, transported, and cultured in the lab further increasing the potential of using peripheral grafts for cell culture [26].

Thus, the results reflect that if both the zones are cultured or if the peripheral zone is cultured and the central is transplanted, almost all the corneas available for transplants have a potential for providing two full grafts from one donor eye, depending on the preliminary endothelial cell counts. This would substantially increase the donor endothelial cell pool for regenerative treatments.

## Figures and Tables

**Table 1:** Cell area, hexagonality, polymorphism, and Ki-67 positivity of the cells cultured from central and peripheral zones.

	Cell area (*μ*m^2^)		Hexagonality (%)		Polymorphism (%)		Ki-67 (%)	
	Central	Peripheral	Central	Peripheral	Central	Peripheral	Central	Peripheral
Average ± standard deviation (range)	407 ± 16.09 (389–420)	404 ± 25.06 (380–430)	69.66 ± 1.53 (68–71)	71.33 ± 4.04 (67–75)	19.67 ± 4.73 (16–25)	21 ± 4.58 (17–26)	7.67 ± 1.53 (6–9)	8.33 ± 1.15 (7–9)
*p* value	0.8713		0.5593		0.7434		0.5813	

## Data Availability

All the data are available in the Veneto Eye Bank Foundation (FBOV, Italy) repository.
